# Human-Building-Technology Interactions in Healthcare Environments: A Guiding Analytical Framework Based on Mediation Theory

**DOI:** 10.1177/19375867251332642

**Published:** 2025-04-30

**Authors:** Jodi Sturge, Wouter Eggink, Omar Martinez Gasca, Geke Ludden, Margo Annemans

**Affiliations:** 1 Department of Design, Production and Management, Faculty of Engineering Technology, 3230 University of Twente, Enschede, The Netherlands; 2 Department of Product Design, 26660 University of Antwerp, Antwerpen, Belgium; 3 Department of Interior Architecture, 26660 University of Antwerp, Antwerpen, Belgium

**Keywords:** design principles, design theory, future interaction, human-centred design, interdisciplinary study, smart healthcare

## Abstract

**Objective:** There is a need for a more theoretical understanding of human behavior to inform the decision-making related to how technology should be integrated into healthcare environments. **Background:** Healthcare systems are transforming with more technology embedded within the built environment of healthcare facilities. The placement of these technologies, however, only sometimes considers the needs or workflow of patients, visitors or staff. Despite similarities, evidence-based design, smart building design and human–building interaction research rarely intersect. However, each relies on multi-disciplinary insights to enhance these fields. In this paper, we contextualize human–building interaction with building technology through an analytical framework inspired by mediation theory. **Methods:** Based on five case examples from previous studies and site visits, we present the interaction and explain how mediation theory provides insight into the interaction. **Results and Conclusions:** Looking at human–building technology interaction from the lens of mediation theory, it is apparent that the specific decisions taken in spatial and technological design impact the behaviors of building occupants. This paper provides examples of how technology in healthcare environments is used unintendedly, resulting in adapting the use to meet the user's needs. Mediation theory provides a framework to contextualize such encounters which will allow researchers to anticipate user needs and avoid disruptive building technologies in the future.

## Introduction

Healthcare buildings, from hospitals to residential care, are complex environments with various functions and user groups, including patients, visitors, and staff. The design of the built environment and the building systems of these environments can both positively and negatively impact health outcomes and working conditions (Jamshidi et al., 2020; Shajahan et al., 2019).

Inspired by the early work of Ulrich (1984), the discipline of evidence-based design (EBD) has evolved as an interdisciplinary, scientific field in which the impact of the built environment on an individual's health is understood (Hamilton, 2020). Several systematic reviews in the field of EBD (Elf et al., 2024; Nilsson et al., 2020; Jin et al., 2023; Rowe & Knox, 2023; Ulrich et al., 2008; Verderber et al., 2021) have focused on the impact that healthcare buildings, various clinical settings, and features have on patient's outcomes and optimal staff functioning. Yet, findings are rarely replicated or applied to inform healthcare environment design with theoretically informed or rigorous empirical research (Taylor, 2020). As found in the review by Elf et al. (2024) of over 400 articles, a limited number of EBD studies are based on a theoretical framework, suggesting a need for researchers to explore theoretical frameworks to enhance the rigor of research, implementation of the findings, and evaluation of results. Comparably, a variety of terminologies have been coined to describe approaches to inform the design of hospital and healthcare environments, including salutogenesis, healing architecture, and smart hospital; however, there are no common definitions or research methods have emerged to translate these terms into practice (Mazzi, 2021; Rajaei et al., 2023; Simonsen et al., 2022).

Subsequently, using “smart” building design (SBD), systems and technology are becoming increasingly common as a cost-effective and efficient way of providing healthcare that supports staff performance and resource optimization (Rajaei et al., 2023; Woll & Tørresen, 2022). For instance, the use of building management systems (BMS) to regulate and interact with the built environment (e.g., airflow, heat, or lighting), security systems to monitor activity, or systems to optimize workflow (e.g., patient check-in systems or sensors). The application of these systems often relates to functional factors of the environment, such as efficiencies, costs, and environmental pillars of sustainability of the building, missing the social element – designing for the needs of people (Buckman et al., 2014). To better understand the dynamics of human interaction with technologies and systems embedded within built environments, human–building interaction (HBI) has emerged. HBI is “an interdisciplinary field that aims to understand how built environments affect human outcomes and experiences and how humans interact with, adapt to, and affect the built environment and its systems” (Becerik-Gerber et al., 2022, p. 2). This approach underscores the importance of a critical understanding of user experiences, routines, and interactions with environments which inspires future designs to be not only “evidence-based” or “smart,” but human-centered and socially sustainable (Lami & Mecca, 2020; Margariti et al., 2023).

EBD, SBD, and HBI all delve into human–environment interaction. However, despite their common ground, these three fields have yet to be explicitly linked. To fully comprehend the intricate interplay between the elements in a (healthcare) environment, a theoretical understanding of human–building technology interaction is crucial. Therefore, the aim of this paper is to explore how a theoretical perspective of healthcare built environment research can provide methodological guidelines and frameworks resulting in a deeper understanding of how to design to support health outcomes for patients, visitors, and staff (Rajaei et al., 2023; Shannon et al., 2020).

### Human-Building Technology Interaction

**Figure 1.**
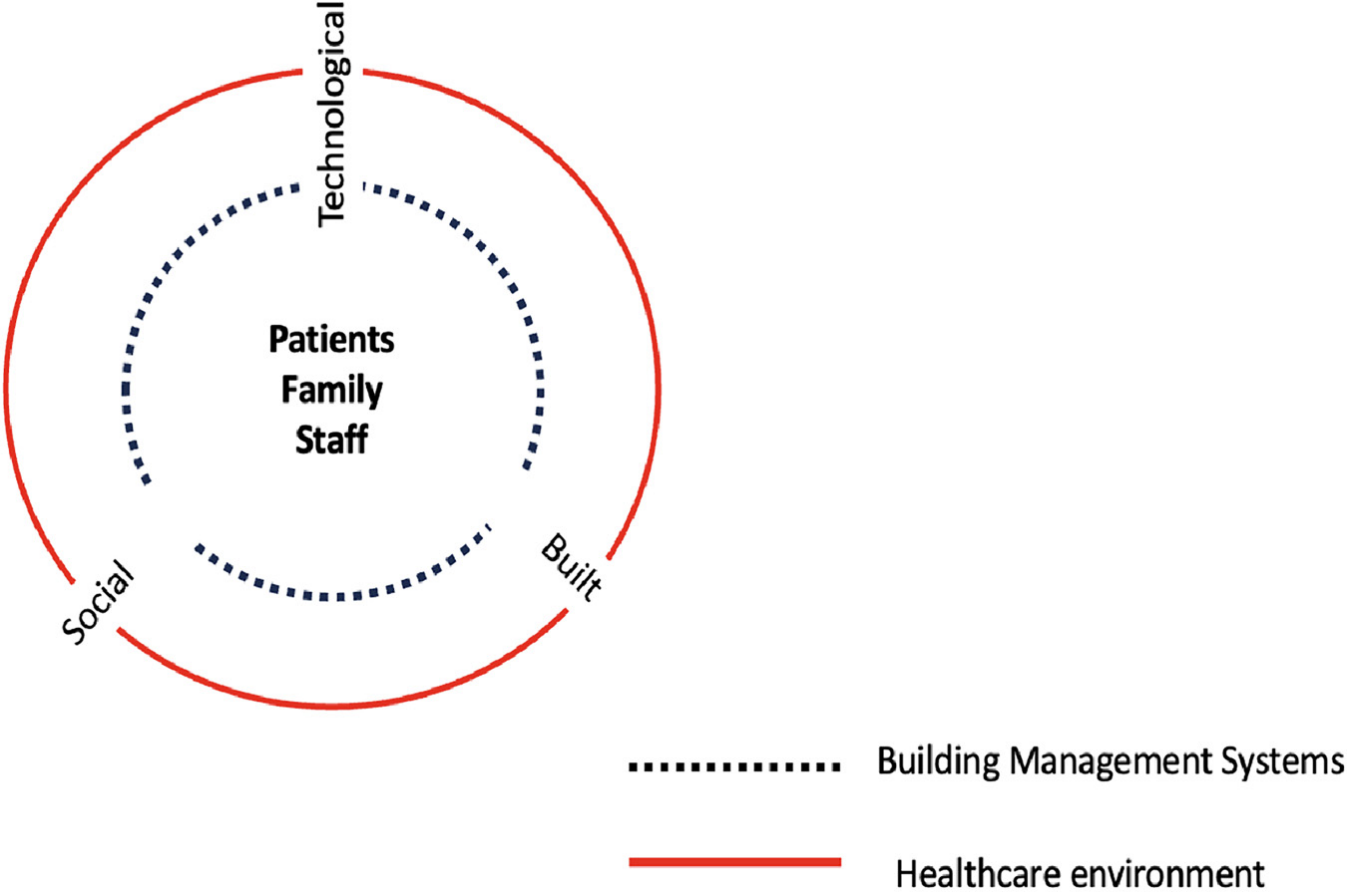
A diagram of the interconnected aspects of a healthcare environment adapted from Alavi et al. (2019).

Conceptualizing human experience, including when and how long people are moving around specific locations, provides a unique perspective on users’ interactions with elements of the built environment (Sturge, 2023). To contextualize the spatial interaction between the different dimensions of the healthcare environment and the importance of a variety of user groups, Figure 1 presents an adapted schema (based on Alavi et al., 2019) in which the aspects of movement between the dimensions within healthcare environments are described. The user groups (i.e., patients, family, and staff) are positioned in the center to demonstrate how they are spatially immersed in a healthcare environment (i.e., the circle) with the notion that the user groups interact and move between the built, social and technological dimensions, which are interconnected. In this context, the built dimension is defined as the physical, human-made structure of the hospital building. The social dimension includes spaces within the hospital where patients, visitors, and staff come together. The technological dimension includes on-site and personal health technologies that monitor personal and patient outcomes, web-based interfaces, and building systems which user groups interact with. Designing and implementing technology in healthcare buildings, based on a theoretical understanding of human–building technology interaction, can result in a multi-disciplinary inspired research-oriented framework that considers the end-user's interaction with the dimensions of the environment (human geography), the building systems, and technology in the environment and the design of the healthcare environments (architecture). Linking theory from human geography, design, and architecture is a promising way to analyze spatial encounters with technological elements of a built environment that account for human behavior. The discipline of human–environment geography provides perspective into the cultural, political, and social conditions that affect interactions with the environment and the dynamics of the architecture building type (Jacobs et al., 2012; Zimmerer, 2017). While design theory explains and understands why a design works (Pries-Heje & Baskerville, 2010).

### Mediation Theory and the Product Impact Tool

To gain theoretical insight into human–building technology interaction, we refer to mediation theory. Originating in post-phenomenology, mediation theory focuses on analyzing the role that technology plays in shaping the relationship between people and their world. Verbeek (2005, 2012) points out how this relation is actively shaped by the perception and action possibilities that technology provides to human beings—as technologies steer people's attention and behaviors in specific ways, they concretize how human beings can be present in the world and how the world can be present to humans. Hence, rather than conceptualizing technologies as an instrumental extension, they must be seen as “the media that establishes our connections to the world” (Verbeek, 2015, p. 29). For instance, let us consider how the proliferation of cameras as an everyday technology embedded in our phones has shaped current notions of photography. This technology has not only caused people to perceive more situations as photographable—as a smartphone allows for quick and efficient access to the camera – its small dimensions make it possible for it to be used in a wide variety of situations, which has also opened a legal and cultural reconsideration of the notions of privacy (Waelen, 2023). Therefore, technologies are not mere instruments through which humans realize their fixed and pre-existing goals. Instead, they guide and steer human intentionality in specific directions through their material, interactive and interpretative qualities (Verbeek, 2015). In this light, technological devices and buildings can be considered “technology.” Mediation theory states that things (e.g., technology) do not need to be “designed” but that the design should be based on the relationship between humans, technologies, and society. However, considering the different forms of relations that humans can establish with technology, it is necessary to translate the analytical concepts of mediation theory into concrete methodological guidelines for design.

**Figure 2.**
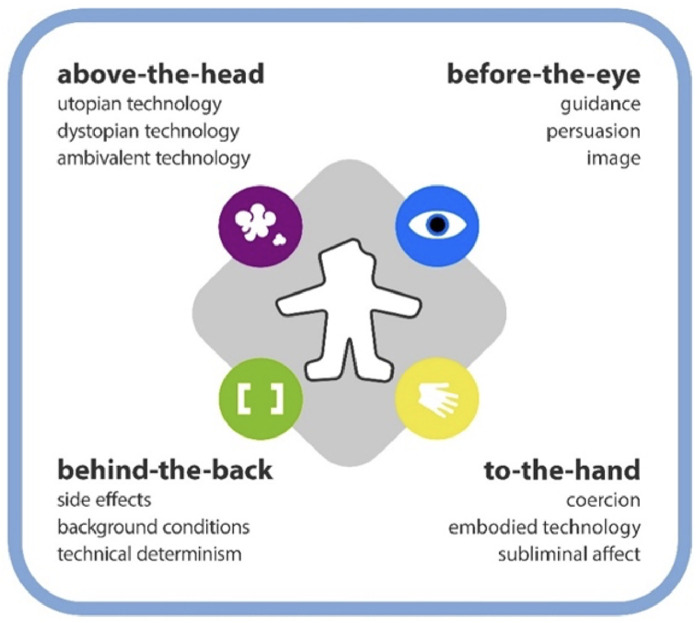
The product impact tool describing the different modes of impact of technology on the user from all sides.

The Product Impact Tool (Figure 2) facilitates the practical application of mediation theory in user-centered design (Dorrestijn, 2017). This tool categorizes the various ways that technologies influence humans into four quadrants: physical and embodied (to-the-hand), visual and cognitive (before-the-eye), invisible and ubiquitous (behind-the-back), and abstract and imaginary (above-the-head). Each quadrant further provides three examples of the modes of influence within that sphere. Such an approach assists designers and decision-makers in anticipating a product's impact on human practices and experiences. However, it also underscores the pressing need to explore the ethical implications of disruptive technologies in healthcare. By examining the compatibility of building feature technology with unanticipated ethical considerations, such as intuitive use, privacy, and adaptability, we can ensure that these technologies serve humanity's best interests (Dorrestijn, 2020; Gračanin et al., 2019; Sadri et al., 2023).

## Method

### Case Selection

Two authors (JS, MA) identified five case examples from previous projects of re-occurring, interactions between users with one or more of the social, built, or technological dimensions of healthcare environments as described in Figure 1. The case examples are based on observations made during fieldwork in previous healthcare research projects (including Annemans et al., 2024) and site visits. These examples highlight the notion that people take up these systems and environments in ways that are different from the intended ones, where the design neglects actual human experience.

These case examples were then presented to two authors (OMG, WE) with expertise in design research and mediation theory. These authors referred to the Product Impact Tool as an analytical framework to assess selected case examples. When analyzing the collected material, they positioned end-users—referring to patients, visitors and staff who occupy built healthcare environments—at the center of care by illustrating how encounters with BMS and healthcare environments impact relations between built, social and technological dimensions (Table 1) in various unintended ways.

**Table 1.** Case Example Details.

Case example	Type of environment	Research activity	Purpose of technology	Relation to dimensions in the schema
Rotating entrance	Hospital	Site evaluation	Heating regulation	Built–technological
Information kiosks	Hospital	Site evaluation	Service efficiency	Social–technological
Heating system	Dementia Care	Observation research	Heating system	Built–technological
Emergency door	Hospital	Observation research	Safety	Built–social
Keypad controlled doorway	Dementia Care	Observation research	Safety	Built–technological–social

## Case Examples

### Case Example 1: Rotating Entrances

**Figure 3.**
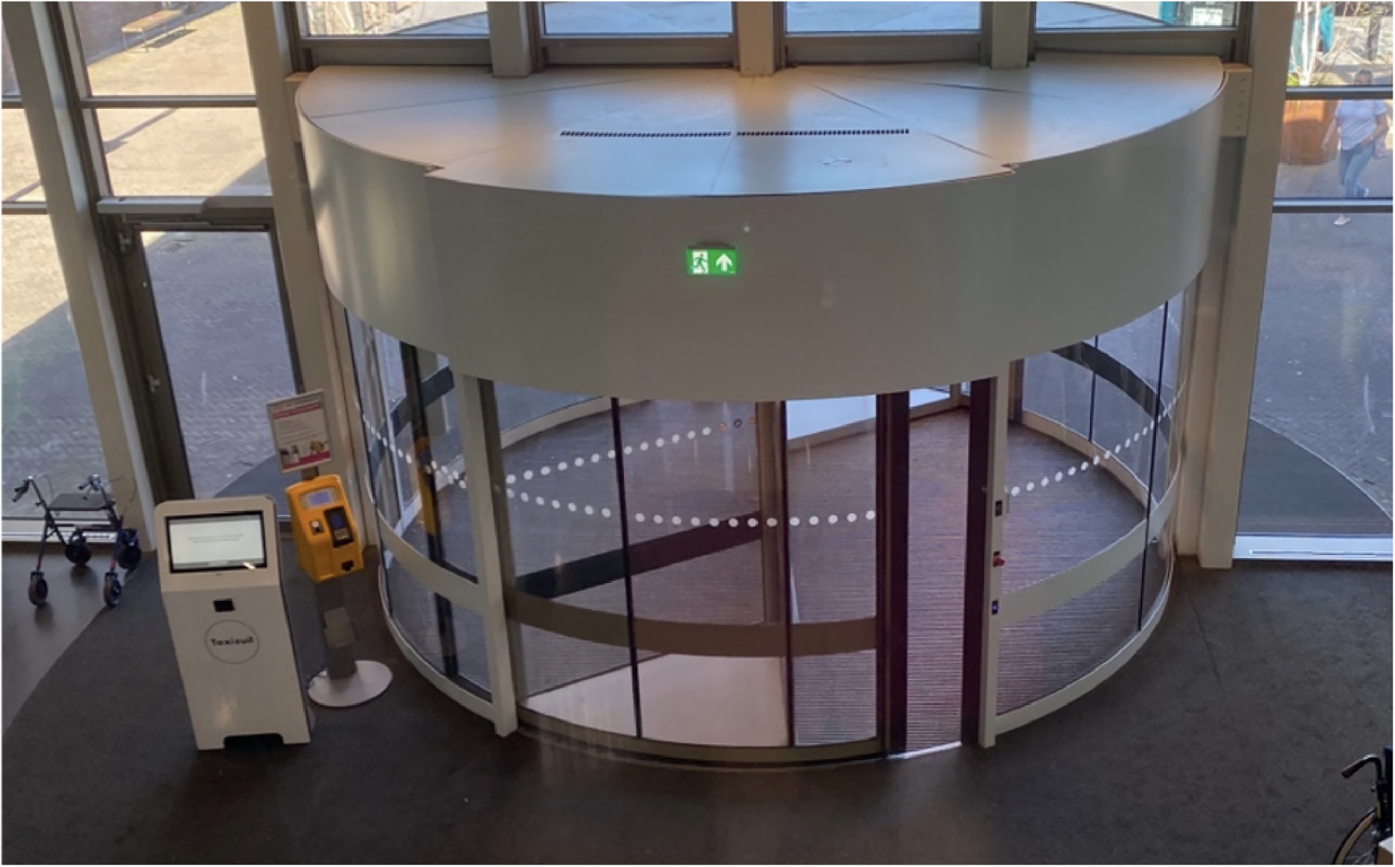
A rotating entrance to a hospital environment.

Automatic revolving doors in hospitals (Figure 3) are a common feature that support the economic and environmental domains of sustainability but less on the social sustainability domain. These mechanical structures are often in place to minimize drafts and save energy costs for the building. However, approaching these doorways is not always an inviting experience, where each model is slightly different in size, capacity, and function. Additionally, these technologies could be more responsive to the speed of the patient or the assistive mobility devices. Sometimes, there is a button for wheelchairs, but not considering other barriers, such as low vision, high stimulation, and a sense of safety.

#### Applying Mediation Theory

Referring to this example, one recognizes the direct physical influences of the doors as belonging to the to-the-hand quadrant. The speed of the doors is a form of coercion, forcing every visitor to move through the doors at a certain pace. Influencing the speed of the doors—although controlled by a physical “wheelchair” button—is, however, a form of guidance in the before-the-eye quadrant. This action requires the user to translate the cognitive meaning of the button towards the (physical) altered speed. Moreover, the designation of the speed control as a “wheelchair button” forms a particular image, such as an association with a certain identity, that is not always desirable.

### Case Example 2: Visitor Information Kiosks

**Figure 4.**
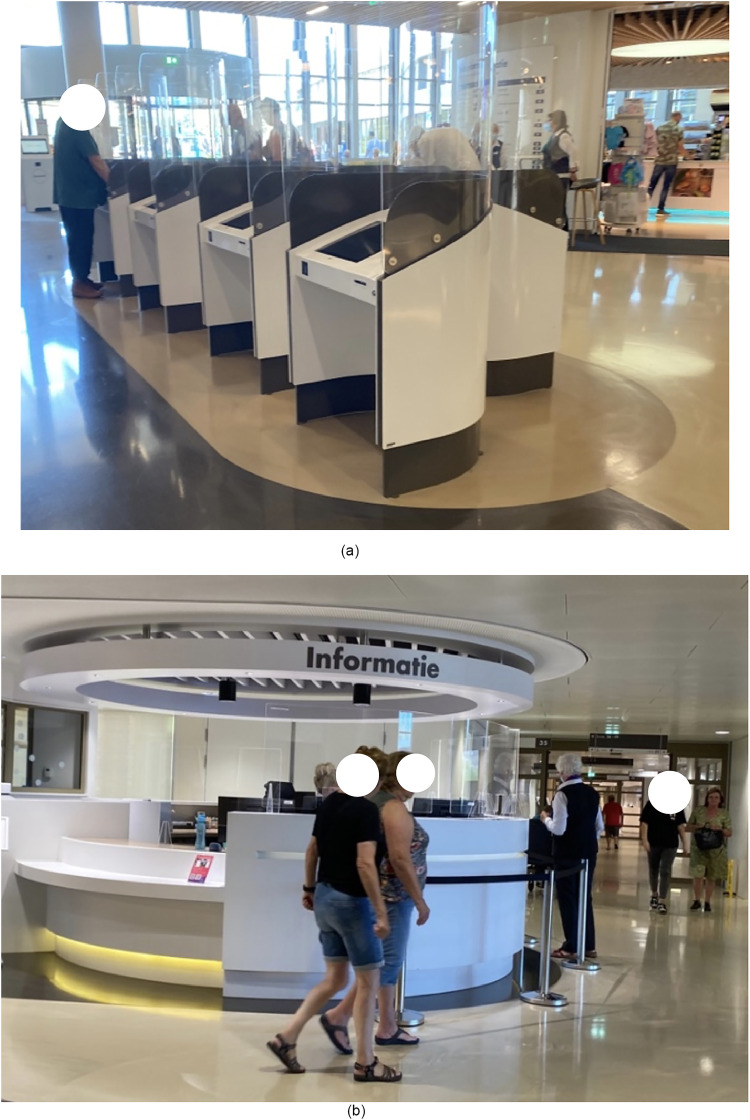
Information and check in kiosks at the entrance of a hospital (picture to the left) and people walking toward an information desk near the entrance of the hospital (picture to the right).

Self-service visitor kiosks (Figure 4) are increasingly common in hospitals. These kiosks are installed to streamline patient check-in and instruct visitors where to go once inside the environment. Here again, economic and efficiency arguments (less personnel and faster check-in) appear to put more weight on the latent social needs of the visitors. However, not all visitors approach these kiosks and instead interact with a human information source at the information desk. This type of interaction could be related to previous experiences that the visitors had, or the design of the kiosks is not inviting, attractive or clear enough.

#### Application of Mediation Theory

The sub-optimal use of the information kiosks (Figure 4) could be seen as a mishap between the envisioned benefits (e.g., efficiency for hospital management) and the perception of the technology by the users. This example is represented by the “above-the-head” quadrant. The hospital management has a utopian view of the implementation of technology that the user can be helped faster and at any time. At the same time, the user might have a more ambivalent or even dystopian view of the technology. They could have a limited understanding of how the interface works or how the hospital will use the data and communicate it to other stakeholders, such as insurance companies.

### Case Example 3: Heating Systems in Corridors

**Figure 5.**
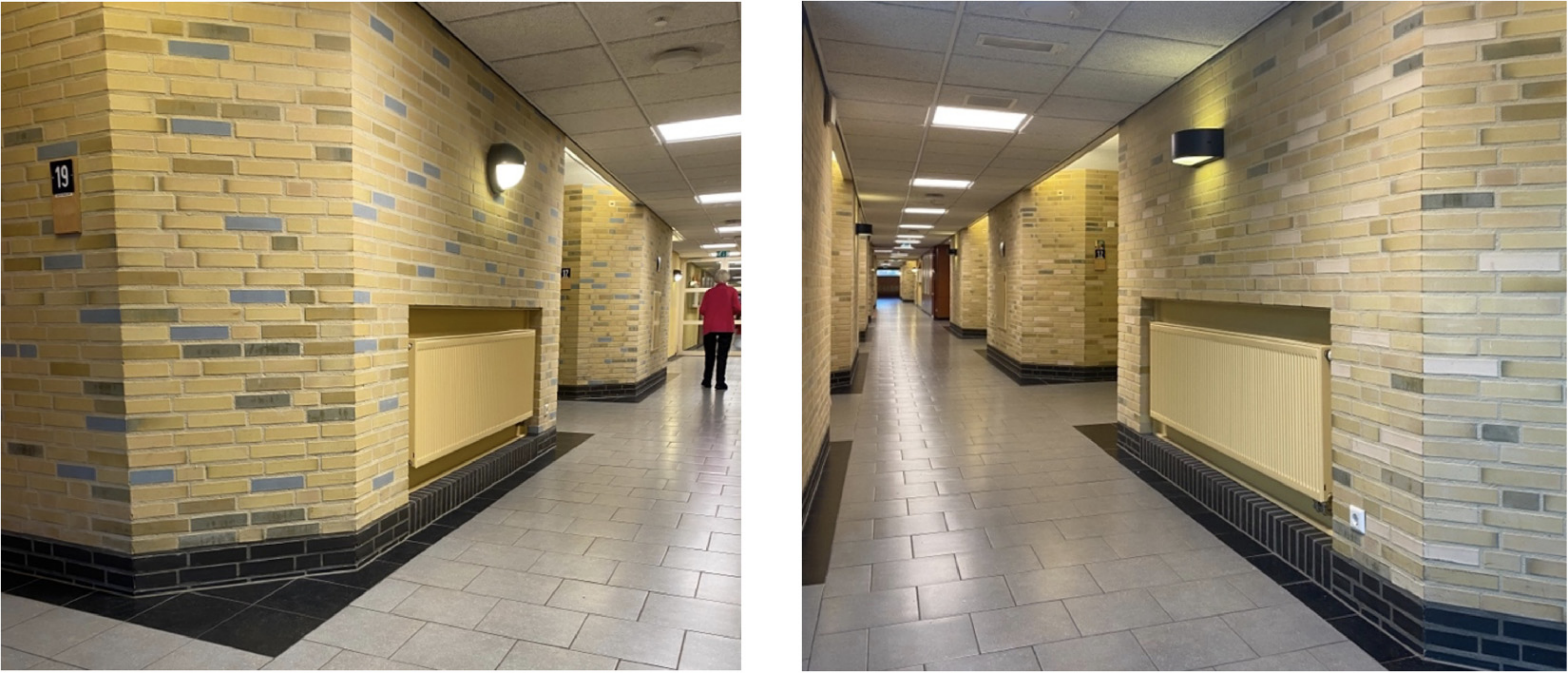
Heaters along the walls of a corridor.

Familiar building technology can provide comfort to patients. As noted during an observation study in a dementia care unit, one resident wandered the corridors repeatedly interacted with a heater on the wall (Figure 5) while pacing through the corridors. The warmth and sensation of the metal grade shifted the relationship to the environment where they resided and appeared to provide a sense of comfort to a resident. This observation is an example of an interaction not intended by the architects of the building, which you can note from the integrated design of the heaters in a niche of the wall. However, these kinds of “unintended use” can play an important role in the actual use of the built environments.

#### Application of Mediation Theory

The observed physical interaction of the client with the heaters of the central heating system as a means of providing comfort is particularly interesting when analyzed with the Product Impact Tool. According to mediation theory, a central heating system is a canonical example of a technology with a background relation to the user (Verbeek, 2005). The system provides its functionality invisibly to the building environment, and the user only indirectly notices the effect: a comfortable, familiar, and uniform temperature. This example is part of the behind-the-back quadrant, described as a background relation. However, for the observed user, the mode of interaction transforms into a physical relation in the foreground, represented by the hand in the lower-right quadrant of the model. The soothing effect of the radiated heat and textured surface could have been foreseen by the designers and anticipated by a more accessible implementation of the radiators in the environment.

### Case Example 4: Emergency Exits

**Figure 6.**
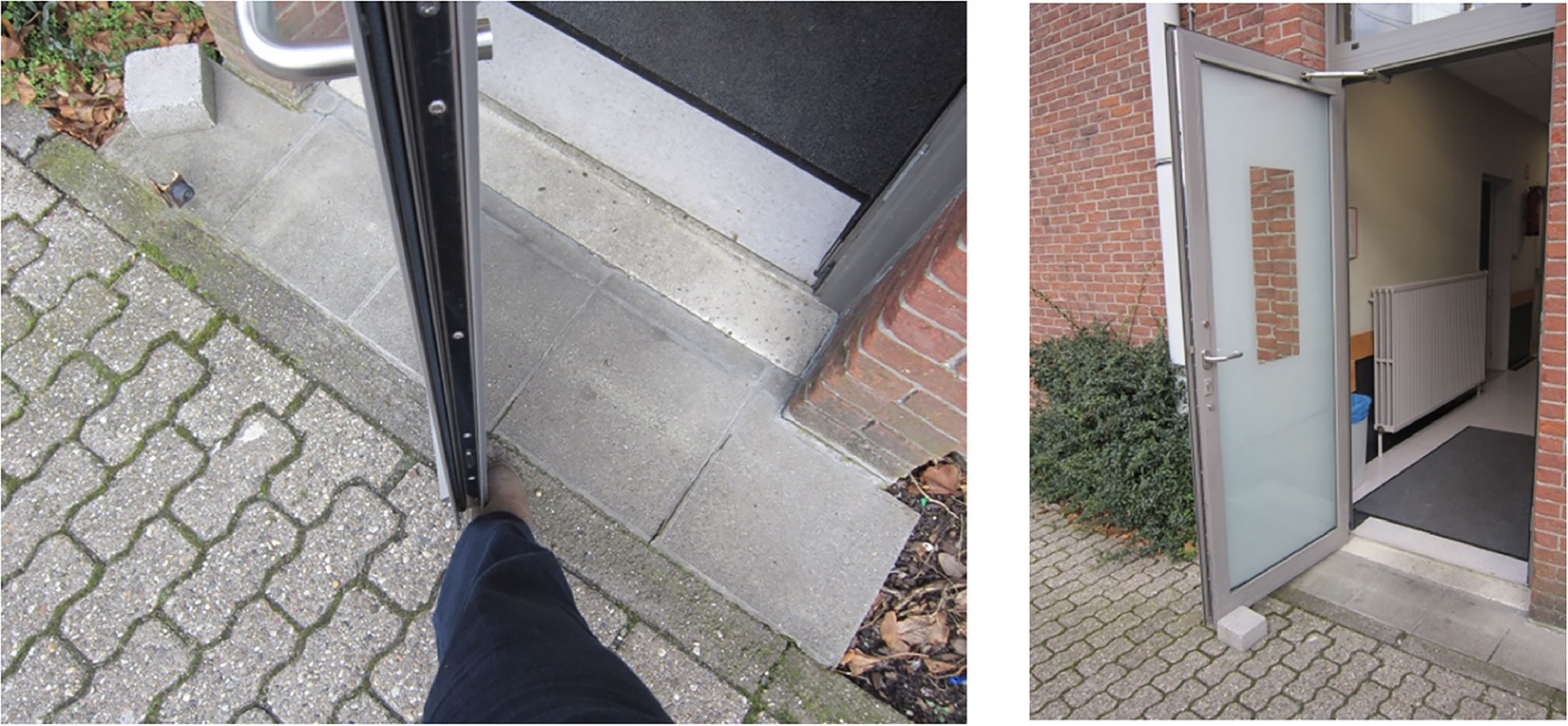
An emergency exit door is held open with a brick.

For safety reasons, emergency exits should always be able to be opened from the inside. For patients who smoke, this provides an excellent opportunity to sneak out to have a cigarette, even at night when the smoker's lounge is closed, to guarantee patients stay inside and rest. Patients place a brick to keep the door open to bypass the automatic closing of this door and being locked outside (Figure 6). Again, this observed interaction identifies a form of unintended use or even misuse that should be considered. On the other hand, it also shows an apparent important need of the patients who smoke that sparks some creativity in circumnavigating the safety measures. The example also shows that the design of these environments is always a negotiation process between the different values of the other stakeholders. The safety (emergency exit) versus healthiness (discouraging smoking) and autonomy (smoking when you want it) versus rest (keeping it quiet at night) (Annemans et al., 2024) dilemma is described in this example.

#### Application of Mediation Theory

Emergency exit doors always open from the inside and can be seen as a form of technological determinism (behind-the-back). The value of safety implies that all emergency doors should be always openable from the inside. Often, signs persuade (before-the-eye) the user not to do that when not in an emergency situation. The “misuse” by the smokers in this example is called a side-effect. This experience causes even another side-effect, as when the smokers circumnavigate the safety measures, they are confirmed in an attitude of disobedience (amplified by the eventual warning signs) that supports the act of smoking in itself as an unhealthy habit.

### Case Example 5: Key Pad Controlled Door

**Figure 7.**
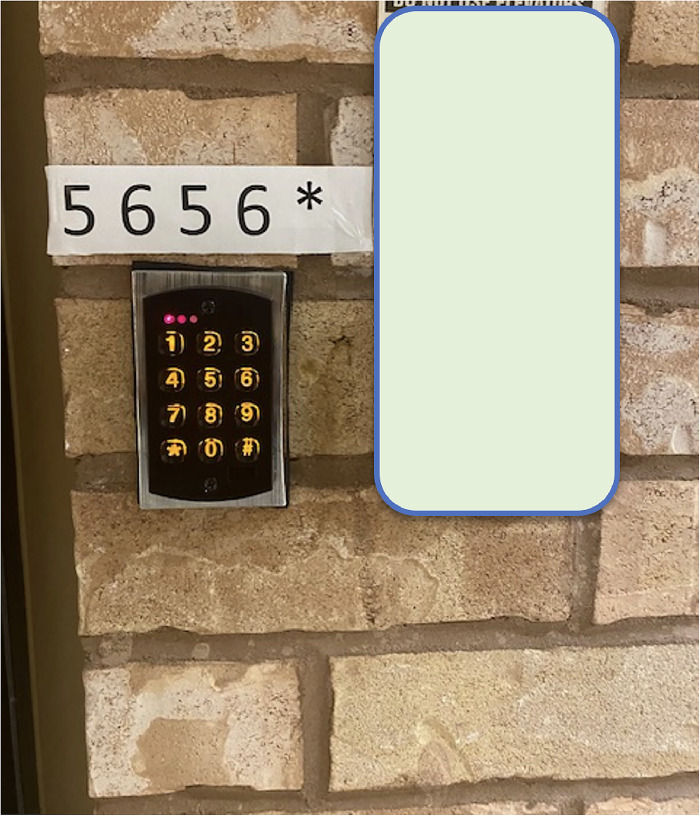
A code to open a secured door is place above a keypad.

To prevent patients with cognitive issues from wandering through or outside the building, doors are often secured. In such circumstances, entrances and elevators can only be used after entering a code on a keypad (Figure 7). To allow visitors, staff, and other patients to use the elevator freely, a paper with this code, written backwards, and the instructions not to let other people into the elevator, is hung up on the elevator. As such, responsibility for patients’ safety, which the technology initially should provide, shifts again to individuals. This results in the opening and closing of the door becoming a topic of discussion, agitation and negotiation (Sturge et al., 2024).

#### Application of Mediation Theory

The keypad at the elevator doors (Figure 7) is an example of what Verbeek calls moral technology (Verbeek, 2006). The device divides users into two groups: people who are cognitively aware enough to read and memorize the instructions and those who are not. As users are not operating the device in a social vacuum, this moral judgement will influence the social interaction in the space around the elevator doors. Often, the people who are not permitted to use the technology are aware of the mechanism of being excluded, which also causes potential discomfort and unease for those allowed to use the elevator. Although the technology might look like operating a simple physical barrier (coercion), it encompasses many issues in the psychological (before-the-eye) quadrant. This case example touches upon similar themes described in examples 1 and 7. On the one hand, it is about how technology can inform our sense of identity (in this case, separating people into two groups) and, on the other, how it establishes the basis for making moral decisions and what can be done to distribute responsibility between people and technology.

## Discussion

*As we transform the future of healthcare around technology.*

As we transform the future of healthcare around technology, the nature of care delivery in terms of workflow, diagnosis, and treatment processes will change. For instance, advanced building systems, technology and the application of machine-learning and AI are examples of tools that will support healthcare transformation to be more energy efficient (Alanne & Sierla, 2022; Aguilar et al., 2021; Bagheri et al., 2021) and socially sustainable (Sun et al., 2023) in the near future. It is crucial to ensure that the implementation of technology does not solely focus on the practical aspects of the built environment, as it neglects the complexity of human–building interaction patterns and the fundamental influence that technology has in shaping an individual's relationship with those spaces (Greenwood & Wolfram Cox, 2023). Therefore, a human-centred understanding of human-building technology interaction will benefit and inspire a broad spectrum of disciplines, including engineering, architecture and health practitioners.

*Therefore, a human-centred understanding of human-building technology interaction will benefit and inspire a broad spectrum of disciplines, including engineering, architecture and health practitioners.*

A promising way to create human-centred care solutions is to apply design research-oriented theory to understand how people interact with healthcare environments. This approach goes beyond using design to “solve user problems” to account for a positive influence and integration of technology into the spatial healthcare environment practices of patients, visitors and staff. A contribution of theory to human–building technology interaction can inform the ethical use and placement of appropriate BMS technologies that respond to users’ needs, which is critical knowledge for designing human-centred design solutions for the future. Observing, assessing, evaluating and correcting such interruptions or unintended use is critical to designing for positive user experiences and well-being. This perspective can inform the “where and why” design decisions can be explored through a variety of methods such as design ethnography (Pink et al., 2020, 2022), occupant participatory research (Lee et al., 2023) or co-design methods to explore possible futures of the integration of building management technologies in built environments (Paraschivoiu et al., 2023).

In this paper, an interdisciplinary team presents mediation theory to guide the implementation of healthcare-building technology that enhances user experience and well-being by considering end-users preferences, safety, and privacy (Lee & Yoon, 2021). Mediation theory situates the end-user in the center to position their needs, experiences, behavior, or social practices at the forefront of human–building technology research in healthcare environments. Such insights reflect the social component of sustainability, which is often overlooked in smart building environments. As demonstrated through the case examples, building technologies can interrupt interaction when facilitated practices do not align with end-user's lived experience and when the function of the building feature or technology does not align with user group practices and experiences. While some utilitarian familiar building elements can provide comfort, as presented in Figure 5.

By categorizing the mechanisms at play in the described situations and how these technologies are adapted to meet user needs unexpectedly, we can better contemplate the implications of the described observations. For instance, as illustrated through the case examples, building technologies are not always used in practice as intended. Constraints imposed by technology (as illustrated in examples 1 and 2) can go beyond the mere issue of usability. These constrained interactions can be the first points of contact with the built environment and technologies which can inform a sense of disability of some users (i.e., persons using wheelchairs or people with cognitive disaabilities or low literacy) (Moser, 2006), which has ethical implications. Further, technology not only influences behavior but normative frameworks as well. In the case of the doors (Figures 3, 6 and 7), a situation arises that is concretized as what “is allowed and what is not,” which ultimately informs the moral decisions that are taken by people in the space. So, it is not only about nudging users into certain actions, it is also about the implications of establishing specific options in the first place. For instance, information kiosks could be a counterpart of “human information sources,” where people may perceive technology differently if only one option existed. Also, what happens when different types of relations to technology “show up to people” based on their own lived experience, and how does technology fit into that dynamic? For example, how people with dementia or autism (De Jaegher, 2013) interact with technology in healthcare settings is likely different than individuals without.

The Product Impact Tool, a practical implementation tool of mediation theory, is used as a framework to analyze case examples. These examples foreground how technology in healthcare environments is not always used as intended, which can result in adapting the use to meet the user's needs, which can have ethical implications. The keypad at the elevator doors (Figure 7) is an example of what Verbeek calls moral technology (Verbeek, 2006). The device divides users into two groups: people who are cognitively aware enough to read and memorize the instructions and those who are not. As users are not operating the device in a social vacuum, this moral judgement will influence the social interaction in the space around the elevator doors. Often, the people who are not permitted to use the technology are aware of the mechanism of being excluded, which also causes potential discomfort and unease for those allowed to use the elevator. Although the technology might look like operating a simple physical barrier (coercion), it encompasses many issues in the psychological (before-the-eye) quadrant. This case example touches upon similar themes described in examples 1 and 7. On the one hand, it is about how technology can inform our sense of identity (in this case, separating people into two groups) and, on the other, how it establishes the basis for making moral decisions and what can be done to distribute responsibility between people and technology.

The benefit of the analyses with the Product Impact Tool for the user-centred design of building technology in healthcare environments is twofold. First, the four quadrants are a means to structure the complex interplay between users and the environment into different modes of interaction with their associated positive, negative, wanted and unwanted effects. Future application of this framework to contextualize end-user routines and behaviors which will allow researchers to ask the right questions in eventual follow-up studies. Secondly, the four quadrants and the sub-division in twelve impact types serve as an inspirational tool for ideating desired future interactions. This approach is especially insightful when combined with scenario-based design (Dorrestijn et al., 2014). The product impact tool provides a framework to anticipate user needs and avoid disruptive building technologies in the future. This insight is critical with future building designs becoming more complex with new forms of interactivity and more embedded technologies. Although these approaches could be of value, even this concept requires insight into the diversity of end-user needs and preferences. This theoretical contribution can prepare designers to consider the placement and implementation when developing building systems, such as automated patient experience technologies, while also supporting a culture of equity and inclusion in care.

## Limitations

Based on the theoretical nature of this paper there are some limitations. One that is the authors did not confirm the Product Impact Tool analysis outcomes plus the case studies are analyzed in a subjective manner and specultative. Further, we are not aware of the modes of interaction which were considered in the initial design of the technology. This background information would make the analysis more rigorous. Secondly, putting patients, family and staff at the center of the design process might impose other blind spots in the healthcare environment, such as environmental instance sustainability. Future research beyond the case examples presented, including observation grid observations and interviews with user groups, will provide further insights into the subjectivity of such interactions. Positioning the results in relation to mediation theory as a method to increase the implementation based on a human-technology perspective in buildings has great potential to conceptualize human experience in healthcare environments and apply theory to research on healthcare environments.

## Conclusion

*Due to the socio-material context of a healthcare setting, how technology is placed in itself can inform how people perceive it.*

Due to the socio-material context of a healthcare setting, how technology is placed in itself can inform how people perceive it. As healthcare environments move toward more automation, detection, and systems for cost efficiencies, understanding human interaction with building technologies is critical to explore. Mediation theory is an example of a theoretical perspective that can lead to a better understanding of effective design solutions in healthcare environments that better reflect user experiences, needs and practices. An interdisciplinary exploration of the needs and experiences of end-user groups can enhance our ability to design more socially sustainable healthcare technology and buildings.

Looking at human–building technology interaction from the lens of mediation theory, it becomes more apparent how specific design decisions can impact the behaviors and outcomes of building occupants. By categorizing the mechanisms at play in the described situations and how these features are adapted to meet user needs unexpectedly, we can better contemplate the implications of the described observations. Applying this framework holds promise for more responsive building design that is human-centred, inviting, engaging and predictive as opposed to “top-down technology developments” (Becerik-Gerber et al., 2022) that tend not to consider the needs, routines and values of end-users. This understanding will not only shape research questions but also steer design decisions (Shannon et al., 2020). Designing and implementing building technologies based on an individual's needs and encounters and correcting interactions will result in developing socially sustainable infrastructure and intelligent buildings with more intuitive interaction between the end-users, making more “healthy” human-building technologies in the future.

## Implications for Practice

- Applying theory to built environment research in healthcare can provide better opportunities for human-centered research, resulting in smarter healthcare environments.
- The connection between the fields of evidence-based design, smart-building design and human-building interaction can provide theoretical understanding of human behaviour and interaction in healthcare environments to inform the decision-making related to how technology -buildings and systems- are integrated into healthcare environments.
- A human-centred understanding of building systems and healthcare environments can benefit and inspire a broad spectrum of disciplines, including engineering, architecture and health practitioners.
